# Desensitization: Overcoming the Immunologic Barriers to Transplantation

**DOI:** 10.1155/2017/6804678

**Published:** 2017-01-03

**Authors:** Supreet Sethi, Jua Choi, Mieko Toyoda, Ashley Vo, Alice Peng, Stanley C. Jordan

**Affiliations:** Comprehensive Transplant Center, Cedars-Sinai Medical Center, Los Angeles, CA 90048, USA

## Abstract

HLA (Human Leucocyte Antigen) sensitization is a significant barrier to successful kidney transplantation. It often translates into difficult crossmatch before transplant and increased risk of acute and chronic antibody mediated rejection after transplant. Over the last decade, several immunomodulatory therapies have emerged allowing for increased access to kidney transplantation for the immunologically disadvantaged group of HLA sensitized end stage kidney disease patients. These include IgG inactivating agents, anti-cytokine antibodies, costimulatory molecule blockers, complement inhibitors, and agents targeting plasma cells. In this review, we discuss currently available agents for desensitization and provide a brief analysis of data on novel biologics, which will likely improve desensitization outcomes, and have potential implications in treatment of antibody mediated rejection.

## 1. Introduction

Kidney transplantation is the treatment of choice for patients with end stage kidney disease as it is associated with improved patient survival, and better quality of life [1, 2]. HLA (Human Leucocyte Antigen) sensitization, resulting from previous pregnancies, blood product transfusions, or previous transplant, and ABO incompatibility pose significant immunologic barriers to kidney transplantation. HLA sensitized patients present vexing problems as they express multiple alloantibodies that often result in crossmatch positivity and hence longer wait times due to the presence of donor-specific antibodies (DSAs). Patients transplanted across these barriers without sufficient desensitization are at high risk for early graft loss from antibody mediated rejection (ABMR). However, those that survive still are at a much higher risk of chronic antibody mediated rejection (CABMR) posttransplantation with decreased overall allograft survival [3, 4]. Approximately 30% of patients on the kidney transplant waitlist in the US are sensitized against HLA antigens, which reduces the opportunities for successful transplantation. With the new kidney allocation system (KAS) giving priority to patients with a calculated panel reactive antibody (cPRA) of 99-100%, there has been an increase in rate of transplants in this group (from 2.3% pre-KAS to around 10% at year one after KAS); however transplants have declined for patients with cPRA 80–94% (10% pre-KAS to 4.9% post-KAS) [5]. Thus, other approaches are needed to improve the access and success of kidney transplants in this disadvantaged group.

To this end, desensitization protocols (probably best termed immunomodulation) emerged in the late 1990s to overcome this humoral incompatibility and optimize the availability of compatible or acceptable donors. The development of novel immunomodulatory therapies (see Table 1) in the last decade has allowed for refinement of desensitization protocols. This emerged in conjunction with better immunological risk stratification with sensitive DSA screening assays and avoidance techniques and has led to improved transplantation rates and favorable short- and long-term outcomes in these high immunological risk patients. This is an important advancement since ESRD patients who remain on dialysis die at high rate while waiting for an allograft [6, 7]. The benefits of desensitization in improving the life expectancy of ESRD patients were shown in a multicenter study of 1025 kidney transplant recipients by Orandi et al. [8]. Patients who received kidney transplants from HLA incompatible live donors had a substantial survival benefit compared to those who waited for HLA compatible transplants from deceased donors or those who did not undergo transplantation at 1, 3, and 8 years (1 yr, 95.0% versus 94.0% versus 89.6, 3 yrs, 91.7% versus 83.6% versus 72.7%, and 8 yrs, 76.5% versus 62.9% versus 43.9% resp., *P* < 0.001 for all comparisons). Our group has also shown that desensitization is cost effective and leads to better patient survival when compared to remaining on dialysis [9].

## 2. Therapeutic Approaches for Immunomodulation of HLA Sensitized Patients

### 2.1. Intravenous Immunoglobulin (IVIg)

The immunomodulatory effects of IVIg were first recognized in the early 1980s when this agent, developed primarily for replacement of humoral immunity, was found to have beneficial effects in autoimmunity and vasculitis [10]. IVIg affects innate and adaptive immune systems, regulating most components of the immune system including antibodies, complements, cytokines, most immune cells, and their receptors [11–13]. Precise mechanism(s) of immune modulation are still not well known although several have been proposed depending on the specific disease. Plasma-derived IgG has since evolved as a critical biologic for replacement therapy in primary and secondary immunodeficiency. Newer manufacturing methodologies based on gentle chromatographic purification have resulted in IgG products expressing higher concentrations and avidities. In addition these formulations are suitable for i.v. (intravenous immunoglobulin, IVIg) or s.c. (subcutaneous immunoglobulin, SCIG) administration [14]. Proof-of-concept studies in the early 1980s in idiopathic thrombocytopenia (ITP) patients [15] were the cornerstone for the use of IVIg/SCIg in autoimmune inflammatory diseases, particularly those mediated by autoantibodies. Labeled autoimmune indications for IVIG include ITP, Kawasaki's disease, Guillain–Barré syndrome (GBS), Chronic Inflammatory Demyelinating Polyneuropathy (CIDP), and Multifocal Motor Neuropathy (MMN); in addition, IVIg has multiple off-label use in autoimmune indications and prevention and treatment of antibody mediated rejection (ABMR) of kidney allografts [10]. Recent data suggest that IVIg can be modified in vitro using tetra-Fc sialylation to produce a candidate drug with 10-fold greater immune modulatory capacity than seen with IVIg [16].

The advantages of IVIg as a desensitizing agent were clearly demonstrated by the only randomized placebo-controlled trial of IVIg conducted by our team through the NIH (1997–2002) [12]. This multicenter study showed improved transplantation rates for highly sensitized patients, 35% in IVIg (2 g/kg monthly × 4 doses before transplant and 4 doses monthly after transplant) versus 17% in placebo; *P* < 0.05. The deceased donor transplantation rates were 31% versus 12% in placebo; *P* = 0.0137, with a graft survival of 80% for IVIg group and 75% for placebo group at 30 months (*P* = NS). Subsequently, Montgomery et al. [6] used a protocol of low dose IVIg (100 mg/kg) and plasma exchange (PLEX) and demonstrated a significant survival benefit for patients with HLA sensitization who underwent living donor transplantation in comparison to patients who remained on dialysis or underwent HLA compatible transplantation. This survival advantage more than doubled by 8 years. Several desensitization protocols using high dose IVIg (2 g/kg) have been described [13, 17].

Today, IVIg remains the cornerstone of all desensitization protocols. Most protocols use either high dose IVIg or low dose IVIg in combination with PLEX. Various mechanisms by which IVIg has been proposed to be beneficial include its inhibitory effect on B cells and T-cell proliferation, upregulation of anti-inflammatory Th2 cytokines and anti-idiotypic blockade of alloantibodies, and enhanced clearance of pathogenic IgG through blockade of FcR receptors [10, 11, 18].

Since these initial studies of IVIg as a desensitizing agent, our group [17, 19] has shown that use of IVIg alone is not sufficient to sustain low levels of anti-HLA antibody and is associated with antibody rebound posttransplantation with antibody mediated rejection (ABMR). In addition, better understanding of the role of cryptic B-cell responses in antibody mediated rejection and decreased allograft survival has led to the acceptance of addition of anti-CD20 monoclonal antibody rituximab to the IVIg protocols for desensitization [20].

### 2.2. Rituximab (Rituxan®)

It is a monoclonal antibody specific for CD20, a member of the membrane-spanning-4-domain A family of proteins. Our group has reported extensively on the efficacy of combination of high dose IVIg and rituximab in lowering anti-HLA antibodies and improving rates of transplantation in the HLA sensitized group [17, 19]. In 79 highly sensitized patients, a combination of IVIg (2 doses of 2 g/kg on day 0 and 30) and rituximab (1 g on day 15) led to significant reductions in T-cell flow cytometry crossmatch from pretreatment (T cell 183.5 ± 98.4 mean channel shifts (MCS) for LD and 162.8 ± 41 MCS for DD) to time of transplant (T cell 68.2 ± 58 MCS for LD [*P* < 0.00006] and 125 ± 49 for DD [*P* = 0.05]), respectively. Time on wait list for DD recipients was reduced from 95 ± 46 months to 4.2 ± 4.5 months after treatment. Twenty-eight patients (37%) experienced acute rejection. Patient and graft survival up to 24 months were 95% and 84%, respectively, with good allograft function at 1 year. The infusions were well tolerated with minimal side effects [21].

Despite good early results from our group, there were questions that arose regarding the efficacy of rituximab in desensitization. To address this directly, we conducted a blinded, placebo-controlled trial of IVIg + placebo versus IVIg + rituximab for desensitization [19]. Shortly after initiation of this study which aimed to have 75 patients entered, we noted 3 SAEs in our first 15 patients entered. When the study code was broken, we noted all SAEs (severe ABMR) were in the IVIg + placebo group. Due to the severe nature of the SAEs and graft losses, we terminated the study with results reported. Although the evaluation of results was limited in this small cohort, clinically important trends were observed where IVIg + rituximab appeared more effective in preventing DSA rebound, ABMR, and development of transplant glomerulopathy determined by protocol biopsies after transplant. Since publication, other reports have demonstrated an important impact of rituximab on anamnestic responses to HLA antigens after transplant [20]. Kohei et al. reported on the benefits of rituximab in preventing early HLA sensitization after transplant in ABOi patients [22]. This intriguing paper showed significant long-term benefits in reducing de novo DSA generation and preventing chronic ABMR compared to a group of nonsensitized living donors that did not receive rituximab. These findings codify the importance of rituximab in controlling allosensitization and prevention of anamnestic responses. These likely represent the most important impact of anti-CD20 therapy in transplantation since there is little evidence that rituximab alone can reduce antibody levels sufficiently to allow for incompatible transplantation [23].

An important question to address is the risk of infectious complications with this protocol. We evaluated infectious outcomes in a retrospective study including 361 patients, 170 of whom underwent desensitization with IVIg and rituximab [24]. No differences were observed in the desensitized and nondesensitized groups. Bacterial infections were similar in both groups, with urinary tract infections accounting for 50%. The rate of viral and fungal infections was similar. However, a trend toward a higher rate of BK viremia was noted in the desensitized group. In another study comparing BKV viremia in desensitized (*n* = 187) and nondesensitized group (*n* = 284), BK viremia was observed in 20% of the desensitized and 10% of the nondesensitized (*P* < 0.001) groups by 2 years after transplant. More patients in the desensitized group had a peak viral load greater than 10,000 copies per milliliter (*P* < 0.001). However, there was no significant difference in BKV-associated nephropathy or graft loss in the two groups. There was an association of BKV viremia with desensitization and lymphocyte induction [25].

To date, other important observations include the absence of cases of progressive multifocal leukoencephalopathy and PTLD in the desensitized patients [26]. A more comprehensive analysis of the impact of desensitization on risk for viral infections after transplant is presented by Dr. Toyoda in this issue.

Loupy et al. [27] described a posttransplant approach to desensitization in deceased donor kidney transplant recipients using high dose IVIg, rituximab and PLEX. This was successful in decreasing DSA, chronic ABMR, and transplant glomerulopathy at 1 year. Renal function was significantly better in this group when compared to the group that received only IVIg after transplantation. Our group has also reported on the significant benefits of IVIg + rituximab versus IVIg alone in improving long-term graft survival [19].

Jackson et al. [28] retrospectively examined posttransplant DSAs in 50 HLA incompatible living donor kidney transplant recipients. All patients received plasmapheresis and IVIg, and those deemed to be at higher immunological risk (multiple transplants, repeated HLA mismatches, and higher CPRA) received rituximab. At 1 month after transplantation, patients who received rituximab had significantly lower numbers of DSAs (*P* = 0.03) and non-DSAs (*P* = 0.003) than those in the control group. The magnitude of the increase in all HLA antibodies at 1 month was also lower in the rituximab group. However, rituximab induction did not significantly impact the persistence of DSA at 1-year posttransplantation (detected in 52% of the patients treated with rituximab versus 40% in the nonrituximab treated group). Importantly, no significant difference was observed in rates of ABMR and in allograft survival at 5 years after transplantation.

Van Den Hoogen et al. [29] conducted a placebo-controlled trial of rituximab as an induction agent for kidney transplant recipients. 280 patients were examined (138 randomized to rituximab and 142 placebo). Overall, no difference in the graft rejection rates was noted. However, when data for high-risk patients (repeat transplants and HLA sensitized) were analyzed, there was a significant reduction in rejection episodes for those who received rituximab (17.9% versus 41.1%, *P* = 0.039). The authors concluded that a single dose of rituximab given as an induction agent at transplant significantly reduces rejection rates in sensitized patients.

A recent prospective study by Shaffer et al. [30] reported 3-year outcomes in 29 highly sensitized patients who were desensitized with high dose IVIg and one dose of Rituximab after transplantation. The study showed a 46% reduction (*P* < 0.001) in the strength of DSA at 1 month after transplant that was sustained throughout the 3-year follow-up period and was observed for both class I and class II DSAs regardless of pretreatment MFI (mean fluorescence intensity) levels. 3-year patient and graft survival were 95% and 90%, respectively, and acute rejection was diagnosed in 4 patients (14%) during the follow-up period. In a Korean study by Hwang et al. [31], patients with high panel reactive antibody (PRA scores) but negative crossmatch who received pretransplant rituximab (*n* = 32) had significantly lower risk of ABMR and higher 3-year graft and patient survival rates (*P* = 0.007 and *P* = 0.037, resp.).

In summary, the use of IVIg + rituximab ± plasma exchange offers acceptable outcomes and improves long-term patient survival compared to remaining on dialysis [9], especially for patients with DSAs of low to moderate intensity. However, Orandi et al. [32] have shown that outcomes of patients transplanted across a complement-dependent cytotoxicity (CDC) crossmatch are unacceptably low and should not be pursued. Thus, the most successful approaches include desensitization and a strategy to avoid offers from donors where the recipient has strong C1q+ DSAs that will likely result in early ABMR and graft loss. In our experience C1q+DSAs are the most difficult to immunomodulate and are the most pathogenic. Thus, limitations do exist. To respond to these deficiencies investigators have developed other approaches [33, 34].

### 2.3. Bortezomib (Velcade®)

It was FDA approved in 2008 for treatment of refractory multiple myeloma and has been successfully used in ABMR treatment in transplantation [35, 36]. Bortezomib inhibits the 26s proteasome which ultimately leads to plasma cell apoptosis. Woodle et al. [37] showed a significant decrease in immunodominant (iDSAs) and successful transplantation in 19 of 44 highly sensitized patients with low acute rejection rates (18.8%) at 6 months, with a protocol incorporating bortezomib, plasmapheresis, and rituximab. Jeong et al. [38] (*n* = 19) used a desensitization protocol of high dose IVIg, bortezomib, and rituximab and demonstrated an increased rate of deceased donor transplantation. These studies were small, open labeled and nonrandomized. Thus interpretation of efficacy is limited. Moreno Gonzales et al. [39] evaluated the efficacy of 32 doses of monotherapy with bortezomib in 10 highly sensitized patients and found only modest reductions in both class I and class II antibodies with no change in cPRA or flow cytometric crossmatch. In addition, the therapy was not well tolerated. Treatment was interrupted or discontinued in half the patients due to symptoms of fatigue, anorexia, insomnia, anemia, thrombocytopenia, peripheral neuropathy, and disseminated varicella zoster in one patient. Currently, the limited data on efficacy and significant AE/SAEs limits enthusiasm for incorporation of this drug into most desensitization protocols. Recent data was presented on the use of a second generation proteasome inhibitor carfilzomib. Currently, only limited data on efficacy are available. However, the posttransplant use of this drug will likely be limited due to nephrotoxicity and risk of thrombotic microangiopathy [40].

### 2.4. Interleukin-6 (IL-6) Receptor Antagonist (Tocilizumab, Actemra®)

IL-6 is a cytokine critical to numerous inflammatory and immunomodulatory pathways and is essential for the maintenance of host defenses [41, 42]. However, excessive and unregulated production of IL-6 result in a number of chronic immune disorders, including a role in the chronic inflammation seen in transplant rejection, patients on dialysis, in crescentic glomerulonephritis, and graft versus host disease (GVHD) [43–45]. IL-6 is one of the major cytokines involved in differentiation of B cells to IgG-secreting plasmablasts and finally to plasma cells [46, 47]. In addition, IL-6 stimulates Th17 cells that increase inflammation and allograft rejection and inhibits the generation of Treg cells [48].

The IL-6R is expressed constitutively only on hepatocytes and some immune cells [49], while soluble IL-6R can bind IL-6 and then transsignal through gp-130 expressed on virtually any cell type. Membrane bound IL-6R signaling is responsible for host defenses while transsignaling likely mediates the pathologic functions of IL-6. Tocilizumab antagonizes both soluble and membrane bound forms of the IL-6 receptor, resulting in inhibition of the classic and transsignaling pathways. Tocilizumab is a humanized monoclonal antibody that is FDA approved for moderate to severe rheumatoid arthritis, systemic juvenile idiopathic arthritis (SJIA), and polyarticular juvenile idiopathic arthritis (PJIA) [50]. Animal models reveal that anti-IL-6 receptor therapy weakens alloantibody responses by increasing Treg and suppressing B-effector and plasma cells in bone marrow [51]. By targeting the IL-6/IL-6R pathway, reduction in antibody production and increases in Treg cells (CD4+, CD25+, FoxP3+) are likely [52]. We recently completed a phase I/II trial of anti-IL-6R therapy for HS patients who failed standard desensitization (IVIg + Rituximab ± PLEX). Patients received IVIg on days 0 and 30 at 2 g/kg and TCZ 8 mg/kg on day 15 and then monthly for 6 months. If transplanted, patients received IVIg once and TCZ monthly for 6 months. With this protocol, 7 of the 10 patients were transplanted and 6-month protocol biopsies showed no evidence of antibody mediated rejection or transplant glomerulopathy. eGFR (MDRD) at 1 year was 60 ± 25 mL/min. DSA strength and numbers were reduced by TCZ at transplant (*P* = 0.024) and 12 months (*P* = 0.0003) after transplantation. The infusions were well tolerated with the most common side effects of elevated blood pressures, thrombocytopenia, and anemia. The results of this small trial are very encouraging and suggest a need for larger randomized controlled trials to determine the overall efficacy of anti-IL6R drug therapy in desensitization [53].

Over the past 5 years, we have studied tocilizumab as an agent to treat chronic ABMR and TG. To date, we compared a group of 37 patients with TG and CABMR treated with tocilizumab, monthly for 6 to 12 months to a historical cohort of 39 CABMR and TG patients treated with IVIg + rituximab. We have noted a significant benefit in graft survival and reductions of immunodominant DSAs as well as stabilization of GFRs over a 5-year observation period. Pretocilizumab and posttocilizumab treatment biopsies performed on selected patients revealed significant improvements in features of ABMR, including decreased C4d + scores and reduced glomerulitis and peritubular capillaritis scores. Although preliminary, these data are encouraging and possibly suggest a role for disruption of IL-6/IL-6R signaling for treatment of CABMR and TG [54].

### 2.5. IgG Endopeptidase (Ides®)

IgG endopeptidase is a bacterial enzyme produced by* Streptococcus pyogenes* that cleaves all four human IgG subclasses at the lower hinge region of IgG heavy chains yielding F(ab′)_2_ and Fc fragments [55]. The rapid inactivation of IgG molecules inhibits both complement-dependent cytotoxicity (CDC) and antibody-dependent cytotoxicity (ADCC). This likely explains the high pathogenic potential of* S. pyogenes* infection which account for more than 500,000 deaths world-wide each year [55]. However, the isolated enzyme could have a large impact on many antibody mediated autoimmune diseases and transplantation. To this end, phase 2 trials are currently taking place in Europe and US, focusing on safety, tolerability, pharmacokinetics, and efficacy of use in chronic kidney disease patients (NCT02224820 and NCT02426684). Initial data are promising, showing complete removal of DSAs prior to incompatible transplantation with prevention of early ABMR. This drug will likely become part of desensitization protocols and may give hope to highly HLA sensitized patients who have received multiple rounds of desensitization without success.

### 2.6. Obinutuzumab (Gazyva®)

It is a humanized anti-CD20 monoclonal antibody that received FDA breakthrough status in November 2013 for the treatment of chronic lymphocytic leukemia (CLL) [56]. It differs from rituximab in that it recognizes the type II epitope of the CD-20 antigen present on the pre-B and mature B cells versus type I epitope recognized by rituximab. In a clinical trial, obinutuzumab was superior to rituximab in B-cell depletion and yielding significantly better outcomes for patients with CLL and non-Hodgkin's lymphoma [57, 58]. Currently, two multicenter clinical trials are taking place in Europe and US to assess safety, efficacy, tolerability, and pharmacokinetics in the highly HLA sensitized chronic kidney disease patients awaiting kidney transplantation (NCT02224820, NCT02475551, and NCT02426684). Obinutuzumab will also likely represent a significant advancement in desensitization and treatment of antibody rejection as it allows for more complete and durable B-cell depletion. It is also the first drug to be brought forward in more than 10 years for potential indication in kidney transplantation and also the first agent being investigated for an FDA indication as a desensitization agent.

### 2.7. Anti-B-Cell Activating Factor (Belimumab, Benlysta®)

Belimumab inhibits growth and differentiation of B cells by blocking B lymphocyte stimulator (also known as BlyS) and is FDA approved for treatment of adults with active systemic lupus erythematous [59]. Belimumab monotherapy was studied as a desensitization agent in kidney transplantation (NCT01025193). However, the study was terminated early for reported lack of efficacy. Currently, a phase 2 double-blinded, randomized, placebo-controlled trial of Belimumab plus standard of care is being examined for prevention of allograft rejection in renal allograft recipients (NCT01536379).

Several other B-cell depleting immunomodulatory agents are currently in pipeline but have not yet been evaluated for use in desensitization.

### 2.8. C5 Inhibitor (Eculizumab®, Soliris) for Prevention of ABMR

The complement system plays an important role in tissue damage, graft dysfunction and loss induced by alloantibodies, and ischemia reperfusion injury [60, 61]. Eculizumab is a monoclonal antibody that binds protein C5 of the complement cascade, inhibiting its cleavage to C5a and C5b and formation of membrane attack complex C5b-9. A recent study by Bentall et al. [62] at the Mayo Clinic reported significantly decreased incidence of early ABMR in 26 highly sensitized recipients with a positive crossmatch against their living donor after treatment with eculizumab. The incidence of ABMR at 3 months was 7.7% (2/26) in the eculizumab group compared to 41.2% (21/51) in the historical control group who received similar plasma exchange based protocol without eculizumab to achieve acceptable crossmatch. The percentage of patients who developed high levels of DSA (MFI > 10,000) in the first three months after transplant was similar in both groups. However, a follow-up on the eculizumab group beyond 1 year reported by Cornell et al. [63] showed similar graft survival rates in the 2 groups at 3 years. A striking finding of the study was the incidence of TG in the anti C5-treated patients who had persistence of DSAs with BFXM > 200 (50% versus 35.7% in control group *P* = 0.75), suggesting other mechanisms for antibody mediated graft injury besides complement activation, that is, ADCC and direct endothelial cell activation. Thus, eculizumab may be helpful in improving short term outcomes in patients who develop low levels of DSA, but in patients with persistent high levels of DSA, the benefits are lost. In addition, a recent multicenter phase 2 clinical trial of eculizumab for prevention of ABMR in HLA sensitized patients (NCT02113891) failed to achieve the primary composite endpoint (defined as the occurrence of biopsy-proven ABMR, graft loss, patient death, or loss to follow-up at week 9 after transplant).

### 2.9. C1 Esterase Inhibitor (Berinert®, C1-INH)

C1-INH is the only plasma protease that regulates the classic complement pathway [64]. C1-INH can also inhibit the mannose-binding serine protease lectin pathway of complement activation. During C1qrs activation by immune complexes, C1-INH can dissociate C1r and C1s from the activated C1 macromolecule, thus preventing proteolytic activation of C4 and C2, blocking the formation of C3 convertase. We recently completed a blinded, placebo-controlled trial of C1-INH for prevention of ABMR in highly HLA sensitized patients. Twenty patients were enrolled in this phase I/II trial and results showed that no patient in the C1-INH group developed ABMR during the 1-month study period [65]. Analysis of complement levels during the study suggested an important inhibitory effect on systemic complement activation and complement activating antibodies by C1-INH. Montogomery et al. evaluated the use of C1 INH as an add-on therapy to standard of care (IVIg/plasmapheresis) for the treatment of ABMR in a multicenter double-blind randomized placebo-controlled pilot study. While there was no statistical difference between groups in the primary end point of posttreatment day 20 histopathology or graft survival, the C1 INH group demonstrated a trend toward sustained improvement in renal function through day 90. Six-month biopsies showed no transplant glomerulopathy (TG) in the C1 INH group (*n* = 7), whereas 3 of 7 placebo subjects had TG [66]. Similar results were seen in a single-arm pilot study from France investigating the potential effects and safety of C1-INH added to high dose intravenous immunoglobulin for the treatment of acute ABMR nonresponsive to conventional therapy (IVIg, rituximab, and plasmapheresis). This small study showed significant improvement in allograft function at 6 months and a decrease in complement C1q-binding capacity of DSA together with reduced C4d deposition in allograft capillaries [67]. These results are encouraging and support the need for larger studies of C1-INH in the prevention and treatment of ABMR.

Both anti-C5 and C1-INH are also being investigated in larger clinical trials for prevention of ischemia reperfusion induced delayed graft function (NCT01756508 and NCT02134314).

### 2.10. Belatacept (Nulojix®)

It is an anti-CD80/CD86 humanized IgG1 conjugate with CTLA4 (CTLA4-Ig) that blocks T-cell costimulation [68]. Belatacept was FDA approved in June 2011 for prevention of rejection in renal transplant patients. Recent data regarding the seven-year follow-up of patients in the belatacept clinical trials revealed a significant benefit of belatacept in reducing de novo DSA generation compared to patients maintained on cyclosporine based therapy (4.6% versus 17.8%) at 7 years [69]. Belatacept has not been examined as a desensitization agent, but data from our animal models suggests CTLA4-Ig is a potent inhibitor de novo DSA generation and also modifies DSA rebound responses [70]. Our study suggests that there may be inhibitory effects of CTLA4Ig on plasma cell IgG production in mice. This could prompt further studies of CLTA4Ig as a desensitization agent.

## 3. Defining Acceptable Crossmatch and DSA Parameters after Desensitization

Several studies have shown that a positive CDC-CXM at time of transplantation is associated with poor outcomes [71, 72]. However, desensitization can reduce alloantibody titers to a level sufficiently low to create an acceptable CXM that allow for transplantation with low-risk for ABMR [73]. In this regard, it is important to recognize that not all DSAs are susceptible to reduction with desensitization. Here, those that are C1q+ and/or have MFI strength ≥ 10,000 are difficult to reduce to an acceptable level. To deal with this, we have adapted a protocol to identify unacceptable antigens as those expected to produce a positive CDC-CXM and when reacted with the sensitized patient's sera [74]. At our center, we define negative CXM by a flow cytometric crossmatch (FCXM) less than 130 mean channel shift (MCS) for B cell and less than 70 MCS for T-cell FCMX after pronase digestion. Pronase treatment is used to remove CD20 from B cells and non-HLA antigens from T and B cells, allowing more precise determinations of HLA specific antibodies and eliminating rituximab effect. DSA binding is determined by the multianalyte bead assay performed on the Luminex platform. The strength of the reactions is graded as weak (<5000 MFI), moderate (5,000–10,000 MFI), and strong (>10,000 MFI). Antibody specificities and strengths are compared to those obtained before desensitization. To simplify analysis, we have created a scoring system to represent MFI intensity of DSAs. The DSA-RIS (relative intensity scale) gives 0 points for no DSA, 2 points for each weak DSA (MFI < 5,000), 5 points for each moderate DSA (MFI 5,000–10,000), and 10 points for each strong DSA (MFI > 10,000) [4, 74]. Desensitization is considered successful if posttherapy donor-specific CXM is acceptable, as determined by negative CDC in 1 : 2 or higher dilution or FCXM with a shift of less than 225 MCSs and DSA-RIS scores ≤17. Using this approach, we have reduced our ABMR rate to ~16% in the first year after transplant.

A critical aspect which deserves to be mentioned here is the absolute need to develop standardized solid phase DSA assays which will allow reproducibility of results over time and from site to site. Establishing standards for reporting and interpreting data is crucial for comparisons of desensitization therapies [75].

## 4. Novel Therapeutics: From Use in Desensitization to Potential Application in Treatment of ABMR

Chronic immunologic injury to the allograft is now recognized as the leading cause of allograft dysfunction and long-term loss [76, 77]. DSAs, which are the target of desensitization strategies, have been implicated in ABMR leading to decreased allograft survival [3, 78, 79]. Hence, advancements in desensitization will potentially have significant implications on ABMR treatment. DSAs can be present before transplant or emerge after transplant in 20–30% patients. These de novo DSAs are mainly class II and associated with a poorer prognosis compared to DSAs to HLA class I [80]. DSAs can damage allografts in several ways including antibody-dependent cellular cytotoxicity, acceleration of graft atherosclerosis, direct endothelial activation, and complement-dependent cytotoxicity, which if untreated can result in rapid loss of the graft [81].

The development of assays to detect complement-fixing DSAs (C1q-DSA Luminex) has provided new insights into the clinical significance of complement-fixing DSAs. Loupy et al. [82] demonstrated a dramatic reduction in long-term allograft survival in patients who developed C1q-DSAs after transplant (HR, 4.78; 95% confidence interval [CI], 2.69 to 8.49). C1q-DSAs in this study also had a higher mean fluorescent intensity score when compared with non-C1q-DSAs indicating a considerable overlap between DSA strength and complement activating capacity. Better understanding of the pathophysiology of ABMR has stimulated interest in development of therapies aimed at depletion of B cells, antibody, and complement inhibition, much like the drugs being studied for desensitization. To date, there are no FDA approved drugs for desensitization or antibody mediated rejection [74].

## 5. Kidney Paired Donation (KPD)

Paired kidney donation offers another opportunity for transplantation of patients who have a living donor but are HLA incompatible. In many circumstances, it provides a good alternative to desensitization. However, patients who are very broadly sensitized with strong binding HLA antibody will still be difficult to match without the use of desensitization therapies. A combination of desensitization therapies with kidney paired donation may allow for a better immunologic match in such cases. Montgomery et al. [83] reported excellent patient and graft survival with no ABMR at a median follow-up of 13 months in 5 highly sensitized patients who received living donor transplants at Johns Hopkins via paired kidney exchange. One of these patients required desensitization prior to kidney transplantation. Yabu et al. [84] reported successful transplantation of five patients with a cPRA of 100% utilizing this hybrid approach. Utilizing high dose IVIg after transplant, Blumberg et al. [85] reported 100% patient and graft survival at a median follow-up of 22 months in 12 highly sensitized patients with DSA (median cPRA 98%) who underwent transplantation via kidney paired program at UCLA. 3 of these 12 patients had ABMR. Several multicenter consortia have been created in an endeavor to increase the donor pool and facilitate multicenter KPD transplants. A key to success of such multicenter programs is a careful assignment of unacceptable antigens in virtual crossmatch [86]. Unfortunately, the very highly sensitized patients are difficult to find matches for even with desensitization.

## 6. Financial Implications of Desensitization

Kidney transplant is well established as the most cost-effective strategy for ESRD patients when compared to long-term dialysis. According to the recent US Renal Data System annual report, the cost of maintaining a patient on dialysis is $84,550/yr, and the cost of uncomplicated transplantation is $29,920 but could increase to $106,000/yr with complicating events [87]. However, with a functioning graft, the annual cost per transplant patient is much lower at $18,000.

When discussing desensitization strategies, it is important to study the impact of these interventions on the overall healthcare expenditure. Our group performed a study assessing the cost/benefit analysis of desensitization with IVIg + rituximab compared with dialysis over a 3-year study period. In the study, 71% of patients were successfully desensitized with IVIg + rituximab at a cost of $28,090 followed by renal transplant (80% LD and 67% DD) at a cost of $92,799 for each patient. The cost of continuing dialysis ($84,639 annually per patient) in 29% percent of sensitized patients who were unresponsive to the desensitization regimen was included in the desensitization arm of the model according to an intention-to-treat analysis. After accounting for the cost of treatment of antibody mediated rejection that occurred in 22% of transplanted patients and cost of return to regular dialysis after graft loss that occurred in 19% patients, the analysis showed $18,753 cost saving in the desensitization arm ($219,914 per patient compared with $238,667 per patient treated in the dialysis arm). This amounts to saving 7.9% of 3-year dialysis patient costs. Most importantly, transplantation was associated with a 14.7–17.6% increased survival compared to conventional dialysis [9].

## 7. Conclusions

The development of desensitization protocols has been a significant advancement in the field of kidney transplantation offering hope for the immunologically disadvantaged group of highly sensitized patients. Despite the recently highlighted successes in desensitization therapies, there is no consensus regarding the need for desensitization and development of new drugs in this area. Clinical trials of novel therapeutic agents are critical to our persistent efforts to increase the longevity of kidney allografts. The collaboration of transplant physicians, immunologists, and pharmaceutical industries is crucial to delineate a path forward to improve access to kidney transplantation.

## Figures and Tables

**Table 1:** Agents of desensitization.

Immunotherapy	Mechanism of action	Dosing
IVIg^*∗*^	Exact mechanism unclear; however some mechanisms include regulation of B-cell antibody production, induction of B-cell apoptosis through FcyR mediated signals, inhibition of dendritic and macrophage cell maturation and function, inhibition of various proinflammatory cytokines, inhibition of complement mediated inflammation	1 g/kg max 70 g daily × 2 doses OR 2 g/kg max 140 g (given over HD) [88]

Rituximab^*∗*^	Anti-CD20	375 mg/m^2^ × Body Surface Area IV over 5–7 hours [89]

Obintuzumab^*∗*^	Anti-CD20	1000 mg IV titrated per package insert

Bortezomib^*∗*^ Carfilzomib^*∗*^	Inhibiting proteasomes	Bortezomib: 1.3 mg/m^2^/dose × 6–8 doses [89] Carfilzomib: 20, 27, 36 mg/m^2^ [90]

Tocilizumab^*∗*^	Anti-IL6 receptor blocker	8 mg/kg (max 800 mg) monthly × 5–7 doses [53]

IgG endopeptidase^*∗*^	Cleaving Igg leaving behind Fc and F(ab′)_2_	0.24 mg/kg IV over 15 minutes [NCT02426684]

Belimumab	Inhibiting binding of B lymphocyte stimulator protein to the B-cell receptors	10 mg/kg IV over 1 hour every 2 weeks for the first 3 doses [88]

Eculizumab^*∗*^	Blocking complement protein C5 and preventing generation of the terminal complement complex C5b-9	1200 mg IV over 1hour then 900 mg IV over 1 hour weekly × 3 doses or more per clinical response [88]

C1 esterase inhibitor^*∗*^	Inactivating complement pathway players C1r and C1s	20 units/kg IV twice weekly × 4 wks [65]

Belatacept	CTLA4-Ig may have potent effects on de novo donor specific antibody generation and plasma cell inhibition	Not used [70]

^*∗*^Immunotherapy agents require premedication with acetaminophen, antihistamine, and glucocorticoid thirty minutes before infusion.
